# Do Negative Symptom Study Volunteers Represent the Wider Schizophrenia Population? Evidence from a Clozapine Clinic

**DOI:** 10.1192/bjo.2026.11146

**Published:** 2026-06-30

**Authors:** Jacob Walsh, Emilio Fernández-Egea

**Affiliations:** University of Cambridge, Cambridge, United Kingdom

## Abstract

**Aims:**

The representativeness of study populations relative to the clinical populations they aim to reflect remains contested. Evidence suggests that some research cohort populations present with less severe symptoms compared to clinical populations. This phenomenon may also occur in studies investigating motivational impairment within schizophrenia, whereby recruited patients have less severe motivational impairment than typical patients. Thus, findings of studies investigating motivational impairments in schizophrenia may not fully generalise to the wider patient population.

We aim to assess the representativeness of participants diagnosed with treatment-resistant schizophrenia who volunteered for a study on motivation compared with patients attending the Cambridge Clozapine Clinic who did not volunteer for that study, across demographics and clinical outcomes, with a specific focus on motivation and pleasure.

**Methods:**

The CHANSS (Characterising negative symptoms in schizophrenia) is a multi-centre international study aiming to characterise the cognitive mechanisms underlying motivational impairment within the negative symptoms of schizophrenia. Data used were collected from the CHANSS study (n=69 clozapine-treated) and from the Cambridge Clozapine Clinic, included in the ethically approved database (n=244 post duplicate removal). Statistical analysis was conducted via R to assess the difference in population demographics (Age, sex, family history, smoking, dose of clozapine) and outcomes (Outcomes were measured using the Positive and Negative Scale (PANSS), Brief Negative Symptom Scale (BNSS), Calgary Depression Scale (CDS) and Global Assessment of Functioning (GAF) Scale). BNSS-MAP, a measure of apathy, was the primary variable studied.

**Results:**

Clinical outcomes followed a non-normal distribution with unequal variance and high levels of heterogeneity. BNSS overall motivation and pleasure score (MAP), as well as its subcomponents of anhedonia, asociality and avolition, showed no significant difference. CHANSS participants taking clozapine had a significantly younger age. The CHANSS cohort showed significantly higher scores across several other symptom measures. CHANSS participants were more likely to have a higher positive score for positive symptom severity, expression, anhedonia, blunting and CDS total score. Power calculations for all significant results returned a high power above (all >0.9).

**Conclusion:**

For the primary outcome of differences in motivation and pleasure, no significant differences occurred between the clozapine-treated CHANSS participants and the Cambridge clozapine clinic cohort. Thus, for studying motivational impairment, the CHANSS Study was successful in recruiting a study population representative of the wider patient population. However, demographic differences and higher symptom scores in domains beyond motivation and pleasure should be considered when interpreting any findings beyond motivational impairment.

